# Factors influencing corneal edema after phacoemulsification combined with intraocular lens implantation in elderly diabetic cataract patients and establishment of a predictive model

**DOI:** 10.1097/MD.0000000000048046

**Published:** 2026-03-20

**Authors:** Maierdanjiang Ainiwaer, Yingying Hong, Binghe Xiao, Li Ning, Yinghong Ji

**Affiliations:** a Eye Institute and Department of Ophthalmology, Eye & ENT Hospital, Fudan University, Shanghai, China; b Key Laboratory of Myopia and Related Eye Diseases, NHC, Chinese Academy of Medical Sciences, Shanghai, China; c Shanghai Key Laboratory of Visual Impairment and Restoration, Shanghai, China.

**Keywords:** corneal edema, diabetic cataract, elderly, intraocular lens implantation, phacoemulsification

## Abstract

This study aimed to explore the factors influencing corneal edema after phacoemulsification combined with intraocular lens implantation in elderly diabetic cataract (DC) patients and to establish a predictive model. A retrospective analysis was conducted on 300 DC patients who underwent phacoemulsification combined with intraocular lens implantation in our hospital between June 2022 and October 2023. Patients were randomly divided into a modeling group (n = 210) and a validation group (n = 90) in a 7:3 ratio. The modeling group was further divided into corneal-edema group (n = 57) and non-corneal-dema group (n = 153) based on the occurrence of postoperative corneal edema. Univariate and binary logistics regression analyses were employed to identify the influencing factors of postoperative corneal edema in DC patients. A predictive model was constructed using SPSS, and the model’s application value was evaluated using receiver operating characteristic curve, calibration curve, and decision curve analysis in R language. There were no statistically significant differences in terms of general information between the modeling group and validation group (*P* > .05). Significant differences were observed in the comparison of diabetes duration, preoperative endothelial cell density, postoperative corneal endothelial cell loss, hypertension, surgery duration, and age between the corneal-edema group and non-corneal-edema group (*P* < .05). The results of binary logistics regression analysis showed that diabetes duration, preoperative endothelial cell density, postoperative corneal endothelial cell loss, hypertension, surgery duration, and age were influencing factors for postoperative corneal edema in DC patients (*P* < .05). The calibration curves of the model in the modeling group and validation group exhibited a slope close to 1, showing good consistency between predicted and actual risks. Receiver operating characteristic analysis results indicated an area under the curve of 0.96 and a standard error of 0.021 (95% confidence interval: 0.872–0.994) in the modeling group and an area under the curve of 0.94 and a standard error of 0.024 (95% confidence interval: 0.867–0.966) in the validation group. Decision curve analysis curve analysis demonstrated that the model had good application efficacy and clinical benefits in both groups. The predictive model based on influencing factors demonstrated good predictive performance and warrants further validation.

## 1. Introduction

Cataracts are a major cause of blindness worldwide, with a high incidence among the elderly population, significantly impacting patients’ visual health and quality of life. Currently, phacoemulsification cataract extraction surgery, with its significant advantages, has become the mainstream treatment for cataracts. This surgery is an advanced and mature ophthalmic technique that involves using an ultrasonic phacoemulsification device through micro-incisional procedures.^[1]^ During the surgery, the ultrasonic handpiece enters the eye through a corneal or scleral incision, releasing ultrasonic energy at the tip to emulsify the opaque lens nucleus and cortex into a milky consistency. Subsequently, using an irrigation and aspiration system that maintains constant intraocular fluid flow, emulsified tissue is aspirated, leaving the posterior lens capsule intact.^[2,3]^ Compared to traditional surgeries, phacoemulsification offers smaller incisions and less trauma, reducing the risk of postoperative astigmatism and enabling faster patient recovery. Additionally, combined with intraocular lens implantation, it can enhance vision and visual quality. However, elderly diabetic cataract (DC) patients have a higher incidence of postoperative corneal edema.^[4]^ Diabetes induces systemic microvascular changes that affect the eyes, leading to corneal nutrient supply disruptions and impairing corneal endothelial cell function and structure. Corneal endothelial cells are crucial for maintaining corneal transparency and physiological function, and their damage increases the likelihood of postoperative corneal edema.^[5]^ Furthermore, in elderly patients, declining physical function and corneal tissue degeneration result in weakened endothelial cell regeneration and repair capabilities, making them less tolerant to surgical trauma. Accurately identifying the influencing factors of postoperative corneal edema in such patients and establishing a predictive model holds significant clinical importance. This process aids physicians in preoperatively assessing risks, devising personalized plans, and implementing targeted postoperative measures. Early identification of high-risk patients through the model can optimize surgeries, enhance care, reduce corneal edema incidence, and improve surgical success rates and postoperative visual recovery outcomes. Therefore, this study aims to explore influencing factors, construct a predictive model, and provide clinical evidence.

## 2. Research objectives and research methods

### 2.1. Research objectives

This study was approved by the Ethics Committee of Eye & ENT Hospital, Fudan University. A retrospective analysis was conducted on 300 DC patients who underwent cataract phacoemulsification combined with intraocular lens implantation surgery at our institution between June 2022 and October 2023. Inclusion criteria: Age-related cataract patients undergoing cataract surgery at our center; patients graded as Emery Little nuclear hardness level III–V; age ≥60 years and <90 years; normal pupil size with a diameter of 7 mm or more upon dilation; normal transparent corneal morphology with a corneal endothelial cell density ≥2000/mm^2^; good fundus condition, with the possibility of exclusion based on postoperative fundus specifics; preoperative examinations ruling out surgical contraindications. Exclusion criteria: Ocular and systemic diseases affecting corneal endothelial cell function; glaucoma; uveitis; history of intraocular surgery or ocular trauma; severe retinal pathologies.

### 2.2. Research methods

#### 2.2.1. Grouping

Patients were randomly divided in a 7:3 ratio using a random number table method into a modeling group (n = 210) and a validation group (n = 90). Within the modeling group, patients were further categorized based on the occurrence of postoperative corneal edema into the corneal-edema group (n = 57) and the non-corneal-edema group (n = 153). Grouping criteria (Xie et al^[6]^ ): Based on the corneal edema grading system, grade 0 indicates transparent non-edematous cornea; grade 1 indicates localized thin misty corneal edema with a smooth endothelial surface and clear iris texture; grade 2 indicates superficial grayish corneal edema with a rough endothelial surface and blurred iris texture; grade 3 indicates diffuse grayish corneal edema with a cracked endothelial surface and unclear iris texture; grade 4 indicates milky white corneal edema with unclear intraocular structures. Postoperatively, any corneal edema level above grade 0 within 1 week was considered as postoperative corneal edema.

#### 2.2.2. Data collection

Patients’ general information and results of routine ophthalmic examinations conducted before cataract surgery were collected using an electronic medical record system. These examinations included visual acuity assessment, slit lamp microscopy examination, intraocular pressure measurement, B-scan ultrasonography, optical coherence tomography (OCT) examination, corneal topography assessment (Pentacam HR, Oculus, Wetzlar, Germany), corneal endothelial microscopy examination (EM-3000, Tomey, Nagoya, Japan), ocular biometry, and intraocular lens power calculation (IOLMaster 700, Zeiss, Oberkochen, Germany), as well as CASIA2 anterior segment OCT (Tomey). Preoperatively, data on best-corrected visual acuity, endothelial cell density, central corneal thickness, peripheral corneal thickness, and lens nucleus hardness were recorded for patients included in the study group.

#### 2.2.3. Postoperative follow-up records

During the cataract phacoemulsification process, the ultrasonic time and cumulative dissipated energy for each patient were recorded, along with any other relevant intraoperative complications. Results calculated and displayed automatically by Centurion phacoemulsification machine for ultrasonic time and cumulative dissipated energy were considered accurate.

**Figure 1.**
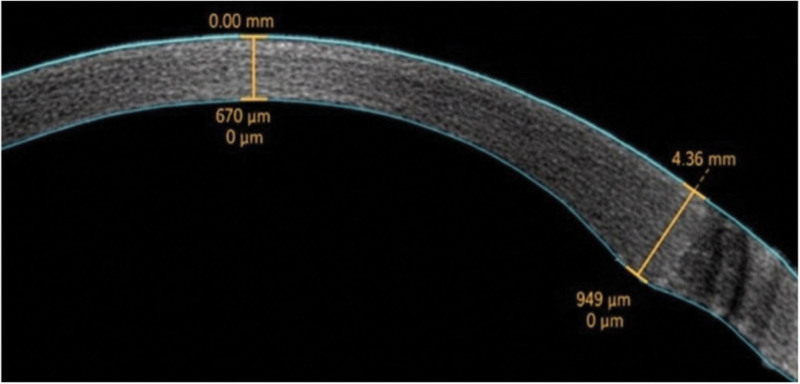
Measurement of incisional corneal thickness by anterior segment OCT. OCT = optical coherence tomography.

On the first day, first week, first month, and third month after the operation, patients returned to the hospital for follow-up visits, which included:

1. Visual acuity and refraction: Under natural pupil size conditions, computerized refraction was first performed to determine spherical and cylindrical powers. Subsequently, subjective refraction was conducted using the RT-5100 ophthalmic refractometer (Nidek, Gamagori, Japan) to measure best-corrected visual acuity. Relevant data were recorded and analyzed using logarithm of the minimum angle of resolution.
2. Slit lamp microscopy examination: Postoperative ocular conditions were observed using a slit lamp microscope. The patient’s corneal status was recorded according to the corneal edema grading system. The position of the intraocular lens and the condition of the posterior capsule were also observed. Any postoperative complications were noted.
3. Corneal topography examination: Corneal scans were performed under dark ambient lighting conditions using the Pentacam HR (Oculus). The instrument automatically scanned the cornea when it was within the scanning area, presenting the results as corneal thickness analysis. Central corneal thickness during postoperative follow-up visits was recorded.
4. Anterior segment OCT examination: Corneal scans were conducted using the CASIA2 anterior segment OCT (Tomey). The instrument automatically scanned the cornea when it was in the scanning area. Manual measurements were adjusted for incisional corneal thickness for each patient, as detailed in Figure 1.
5. Corneal endothelial microscopy examination: Corneal endothelial microscopy examinations were performed using the EM-3000 automatic focusing specular microscope (Tomey). The instrument automatically identified the central corneal region, scanned it, and recorded the corneal endothelial cell density. Three measurements were taken for each patient, and the average value was recorded.

### 2.3. Statistical analysis

The experimental data collected were analyzed using SPSS 27.0 (International Business Machines Corporation, Armonk). The Shapiro–Wilk test was employed for normality testing. For metric data that followed a normal distribution, results were expressed as $\bar{X}\pm S$. Independent sample *t* tests were used for comparisons, while multiple group comparisons utilized *F* tests. Count data were presented as frequencies or rates, and comparisons were made using either χ^2^ test or Fisher exact test. Univariate and binary logistics regression analyses were conducted to identify factors influencing postoperative corneal edema in DC patients. A predictive model was constructed using SPSS, and R language was employed to create receiver operating characteristic (ROC) curves, calibration curves, and decision curve analysis (DCA) to assess the model’s application value. A significance level of *P* < .05 was considered statistically significant for differences. To ensure data integrity, the electronic medical record system was queried for complete data entry. Patients with any missing data for the analyzed variables (including follow-up data at any time point) were excluded prior to the random grouping process, resulting in a complete-case analysis dataset with no missing values requiring imputation.

## 3. Results

### 3.1. General information

Comparison of general information between the modeling group and the validation group showed no statistically significant differences (*P* > .05; see Table 1 for details).

**Table 1:** Comparison of general information between modeling group and validation group.

Baseline data	Modeling group (n = 210)	Validation group (n = 90)	*t/*χ^2^	*P*
Age (yr)	65.13 ± 8.33	66.82 ± 8.51	1.600	.111
BMI (kg/m^2^)	22.41 ± 1.82	22.18 ± 1.94	0.983	.326
Gender				
Male	105	47	0.125	.724
Female	105	43		
Smoking				
Yes	77	31	0.135	.713
No	133	59		
Alcohol drinking				
Yes	88	37	0.016	.898
No	122	53		
Duration of diabetes (yr)	6.93 ± 1.10	7.11 ± 1.08	1.306	.193
Preoperative BCVA (log MAR)	0.95 ± 0.29	0.94 ± 0.31	0.268	.789
Preoperative ECD (cells/mm^2^)	2611.88 ± 264.25	2626.82 ± 271.52	0.445	.657
Preoperative central CCT (μm)	529.01 ± 36.42	533.08 ± 37.52	0.879	.380
Preoperative peripheral CCT (μm)	751.20 ± 44.12	754.97 ± 46.82	0.666	.506
Cumulative dissipated energy	13.92 ± 5.22	13.49 ± 3.98	0.699	.485
Mean ultrasonic time (min)	54.61 ± 21.85	53.84 ± 20.08	0.286	.775
Postoperative ECD loss				
≥10%	40	20	0.397	.529
<10%	170	70		
Corneal malnutrition				
Yes	23	13	0.728	.394
No	187	77		
Hypertension				
Yes	72	36	0.893	.345
No	138	54		
Surgery duration (min)	10.53 ± 1.17	10.32 ± 1.28	1.385	.167

BCVA = best-corrected visual acuity, BMI = body mass index, CCT = central corneal thickness, ECD = endothelial cell density, log MAR = logarithm of the minimum angle of resolution.

### 3.2. Univariate analysis of factors influencing postoperative corneal edema in DC patients

Statistically significant differences were observed in the comparison of diabetes duration, preoperative endothelial cell density, postoperative endothelial cell loss, hypertension, surgery duration, and age between the corneal-edema group and non-corneal-edema group (*P* < .05; see Table 2 for details).

**Table 2:** **Univariate analysis of factors influencing postoperative corneal edema in DC patients**.

Baseline data	Corneal-edema group (n = 57)	Non-corneal-edema group (n = 153)	*t/*χ^2^	*P*
Age (yr)	75.24 ± 8.91	61.36 ± 9.07	9.909	<.001
BMI (kg/m^2^)	22.54 ± 1.73	22.37 ± 2.12	0.542	.589
Gender				
Male	29	76	0.024	.877
Female	28	77		
Smoking				
Yes	19	58	0.374	.541
No	38	95		
Alcohol drinking				
Yes	22	66	0.352	.553
No	35	87		
Duration of diabetes (yr)	8.54 ± 1.12	6.33 ± 1.08	13.055	<.001
Preoperative BCVA (log MAR)	0.93 ± 0.39	0.96 ± 0.42	0.469	.639
Preoperative ECD (cells/mm^2^)	2527.63 ± 267.8	2643.36 ± 231.75	3.082	.002
Preoperative central CCT (μm)	533.51 ± 36.72	527.3 ± 36.12	1.103	.271
Preoperative peripheral CCT (μm)	753.36 ± 46.33	750.37 ± 42.58	0.442	.659
Cumulative dissipated energy	14.16 ± 5.14	13.83 ± 8.31	0.280	.780
Mean ultrasonic time (min)	55.19 ± 25.71	54.39 ± 27.46	0.191	.849
Postoperative ECD loss				
≥10%	17	23	5.893	.015
<10%	40	130		
Corneal malnutrition				
Yes	8	15	0.762	.383
No	49	138		
Hypertension				
Yes	27	45	5.943	.015
No	30	108		
Surgery duration (min)	11.39 ± 1.22	10.21 ± 1.39	5.648	<.001

BCVA = best-corrected visual acuity, BMI = body mass index, CCT = central corneal thickness, DC = diabetic cataract, ECD = endothelial cell density, log MAR = logarithm of the minimum angle of resolution.

### 3.3. Binary logistics regression analysis of factors influencing postoperative corneal edema in DC patients

The significant variables identified in the univariate analysis were assigned as independent variables and analyzed, as shown in Table 3. Postoperative corneal edema (occurring = 1, not occurring = 0) was considered the dependent variable. The results of the binary logistics regression analysis revealed that the duration of diabetes, preoperative endothelial cell density, postoperative endothelial cell loss, hypertension, surgery duration, and age are factors influencing postoperative corneal edema in DC patients (*P* < .05), as shown in Table 4.

**Table 3:** Variable assignment.

Influencing factor	Assignment
Duration of diabetes	Original value
Preoperative endothelial cell density	Original value
Postoperative endothelial cell loss	<10% = 0, ≥10% = 1
Hypertension	No = 0, yes = 1
Surgery duration	Original value
Age	Original value

**Table 4:** Binary logistics regression analysis results.

Variable	*B*	SE	Wald	*P*	Exp(*B*)	95% CI	
						Lower limit	Upper limit
Duration of diabetes	0.771	0.315	5.991	<.001	2.162	1.166	4.009
Preoperative endothelial cell density	0.670	0.248	7.296	<.001	1.954	1.202	3.177
Preoperative endothelial cell density	0.776	0.307	6.383	<.001	2.172	1.190	3.964
Hypertension	0.511	0.232	4.852	<.001	1.667	1.058	2.627
Surgery duration	0.459	0.228	4.047	.001	1.582	1.012	2.473
Age	0.813	0.334	5.921	<.001	2.254	1.171	4.338

CI = confidence interval, SE = standard error.

### 3.4. Establishment of predictive model

**Figure 2.**
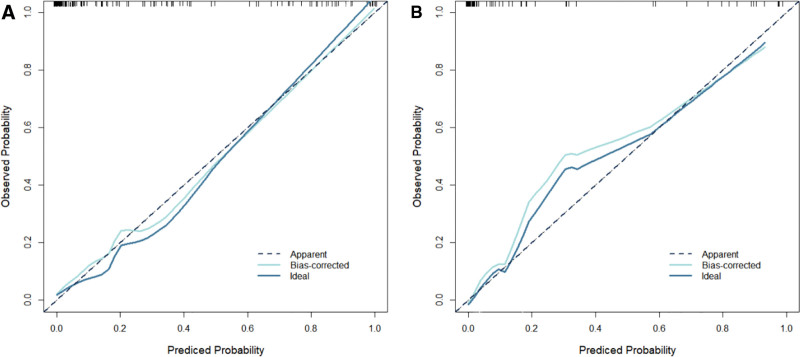
(A) Represents the calibration curve for the modeling group and (B) represents the calibration curve for the validation group. The curves are close to a slope of 1, indicating good consistency between the predicted risk and the actual risk.

Based on the results of the logistics regression analysis, variables including duration of diabetes, preoperative endothelial cell density, postoperative endothelial cell loss, hypertension, surgery duration, and age were incorporated into the constructed predictive model. The combined predictive model expression was determined as Logit(*P*) = 2.948 + (0.771 * Duration of Diabetes) + (0.670 * Preoperative Endothelial Cell Density) + (0.776 * Postoperative Endothelial Cell Loss) + (0.511 * Hypertension) + (0.459 * Surgery Duration) + (0.813 * Age). In both the modeling group and validation group, the calibration curve of the model exhibited a slope close to 1, indicating a good consistency between the predicted risk and the actual risk (see Fig. 2 for details).

### 3.5. ROC curves

**Figure 3.**
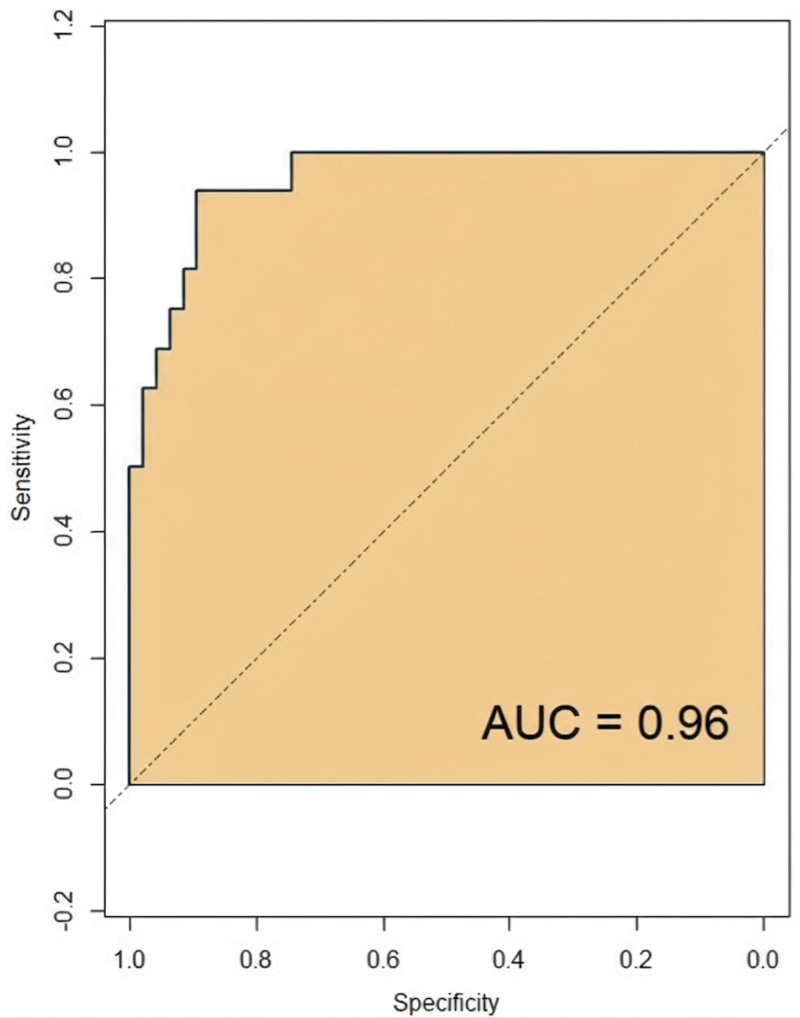
ROC curve of modeling group. AUC = area under the curve, ROC = receiver operating characteristic.

**Figure 4.**
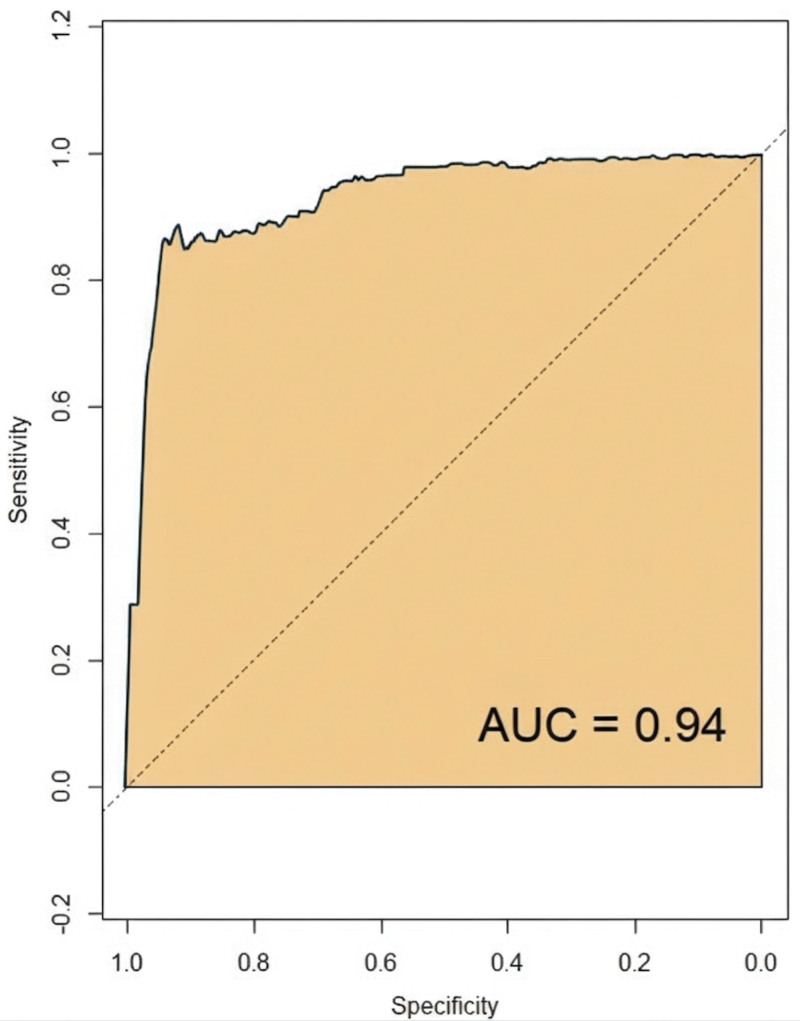
ROC curve for the validation group. AUC = area under the curve, ROC = receiver operating characteristic.

The ROC analysis results indicated that the model’s predictive curve area under the curve was 0.96 in the modeling group, with a standard error of 0.021 (95% confidence interval: 0.872–0.994) and a Youden index of 0.81. At this point, the sensitivity was 92.54%, and the specificity was 88.37%, as shown in Figure 3. In the validation group, the model’s predictive curve area under the curve was 0.94, with a standard error of 0.024 (95% confidence interval: 0.867–0.966) and a Youden index of 0.75. At this threshold, the sensitivity was 81.21%, and the specificity was 93.33%, as depicted in Figure 4.

### 3.6. Clinical benefit analysis of predictive model (two figures)

**Figure 5.**
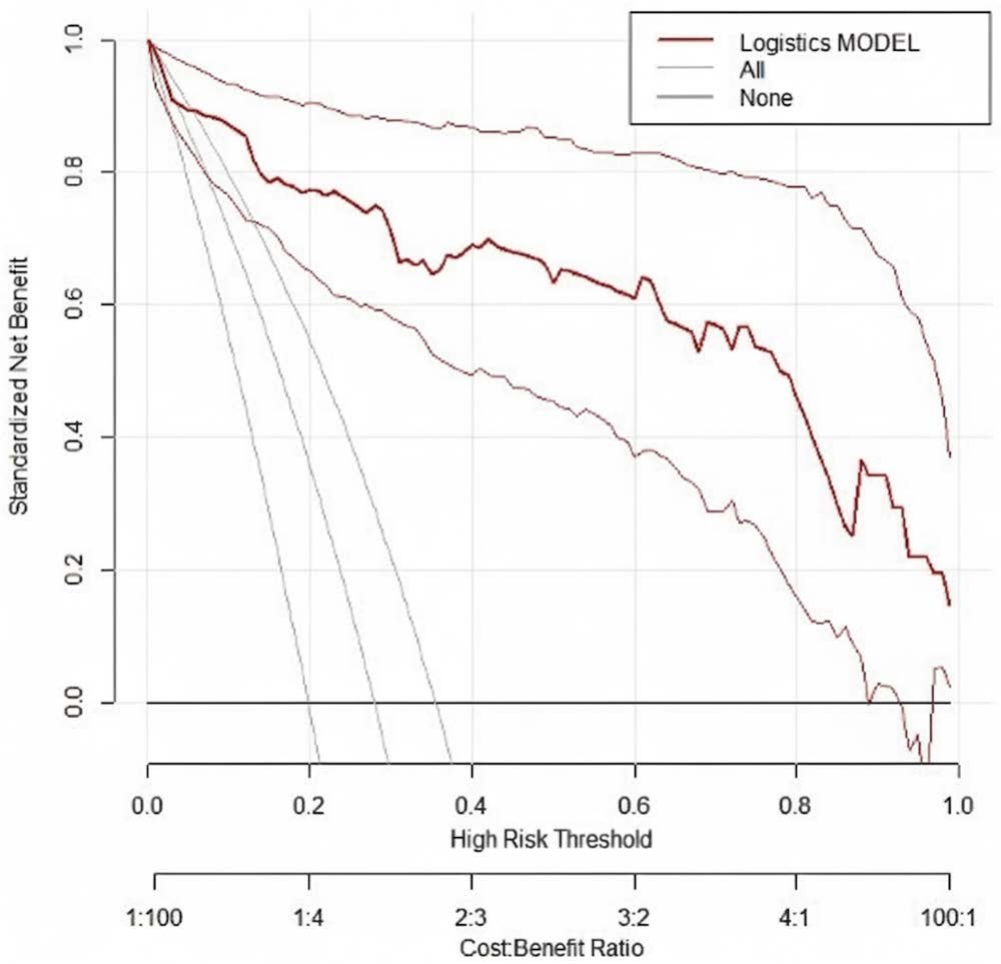
DCA curve. DCA = decision curve analysis.

Drawing DCA curves to evaluate the clinical utility of the model in predicting therapeutic efficacy, the “net benefit rate” balances the benefits of accurately treating high-risk patients against the harms of overtreatment in low-risk patients. In the DCA curve graph, the *Y*-axis represents the net benefit rate for patients following clinical decisions guided by the model. The red curve is plotted based on the model’s predicted target population compliance, while the black and gray horizontal lines represent the extreme scenarios of no patients and all patients developing corneal edema, respectively. The closer the red curve is to the black and gray lines, the lower the clinical value of the model. Decision curve analysis curve analysis in this study demonstrated that the model performed well in both the modeling group and validation group, providing good clinical benefits (see Fig. 5 for details).

## 4. Discussion

This study focuses on the factors influencing postoperative corneal edema in elderly patients with DC combined with intraocular lens implantation and the establishment of a predictive model. Through a retrospective study of 300 patients, they were divided into a modeling group (210 cases) and a validation group (90 cases). Furthermore, the modeling group was subdivided into a corneal-edema group (57 cases) and a non-corneal-edema group (153 cases). The results revealed significant differences between the corneal-edema group and non-corneal-edema group in terms of duration of diabetes, preoperative endothelial cell density, postoperative endothelial cell loss, hypertension, surgery duration, and age. Binary Logistics regression analysis indicated that these factors are all associated with postoperative corneal edema. The predictive model constructed based on these findings showed a calibration curve slope close to 1, and both ROC analysis and DCA curve analysis demonstrated the model’s good performance in the modeling and validation groups, indicating its clinical utility.

Duration of diabetes is one of the key factors affecting postoperative corneal edema in elderly patients with DC. Prolonged hyperglycemia can lead to microvascular changes in the eye, affecting corneal nutrition.^[7]^ As the duration of diabetes increases, microvascular changes in the eye worsen, leading to varying degrees of metabolic and functional damage to corneal endothelial cells. Corneal endothelial cells are crucial for maintaining corneal transparency and normal physiological function, and their dysfunction increases the risk of corneal edema.^[8]^ In this study, patients in the corneal-edema group had longer durations of diabetes compared to the non-corneal-edema group, further confirming the association between diabetes duration and postoperative corneal-edema. Hypertension is also a contributing factor to postoperative corneal-edema in elderly patients with DC. Hypertension can lead to vascular changes in the eye, endothelial dysfunction, and increased intraoperative blood pressure fluctuations, increasing the risk. The study found a higher proportion of hypertensive patients in the corneal-edema group compared to the non-corneal-edema group, indicating a close relationship between hypertension and postoperative corneal-edema. Previous studies by Li et al^[9]^ have also demonstrated the association of diabetes and hypertension with postoperative corneal edema, further corroborating the results of this study.

With advancing age, the physiological functions of elderly patients gradually decline, leading to a series of physiological changes in corneal tissues. The regenerative and repair capabilities of corneal endothelial cells diminish, reducing their resistance to various damaging factors.^[10]^ Additionally, elderly patients often have multiple systemic diseases such as cardiovascular and metabolic disorders, which further impact the ocular health.^[11]^ In this study, patients in the corneal-edema group were older than those in the non-corneal-edema group, indicating age as a significant influencing factor for postoperative corneal edema. Preoperative endothelial cell density is a crucial indicator reflecting the functional status of corneal endothelial cells. Corneal endothelial cells play a pivotal role in maintaining corneal dehydration and transparency due to their pumping function.^[12]^ In elderly patients, corneal endothelial cell count gradually decreases with age. Moreover, in diabetic patients, factors like metabolic disturbances lead to more severe damage to corneal endothelial cells, resulting in lower preoperative endothelial cell density.^[13]^ When endothelial cell density is too low, their pumping function weakens, leading to ineffective removal of moisture from the cornea, thereby increasing the likelihood of postoperative corneal edema. The study results indicated that patients in the corneal-edema group had significantly lower preoperative endothelial cell density compared to the non-corneal-edema group, suggesting that preoperative endothelial cell density can serve as an important indicator for predicting postoperative corneal edema. Prior research by Jing et al^[14]^ has also demonstrated the association between older age, lower corneal endothelial cell density, and postoperative corneal edema, further supporting the findings of this study.

During the process of phacoemulsification combined with intraocular lens implantation, surgical procedures can potentially cause a certain degree of damage to corneal endothelial cells, leading to postoperative loss of endothelial cell count. Factors such as mechanical stimulation from surgical instruments, vibration and heat from ultrasound probes, and impact from irrigation solutions can all disrupt the structure and function of corneal endothelial cells.^[15]^ Excessive postoperative loss of corneal endothelial cells further diminishes their pumping function, impeding the maintenance of normal corneal dehydration and consequently triggering corneal edema. Additionally, the duration of surgery also influences postoperative corneal edema. Prolonged surgical duration implies increased exposure of the cornea to the surgical environment, extending the time of exposure to stimuli such as surgical instruments and ultrasound energy, thereby increasing the likelihood of corneal endothelial cell damage.^[16,17]^ Moreover, prolonged surgical procedures can lead to increased intraocular fluid leakage, exacerbating the degree of corneal edema. The results of this study indicated that patients in the corneal-edema group had longer surgical durations than those in the non-corneal-edema group, with a higher proportion of patients experiencing a loss of ≥10% in corneal endothelial cell count compared to the non-corneal-edema group. Previous research by Chen et al^[18]^ has also demonstrated the association between corneal endothelial cell loss and postoperative corneal edema, further supporting the findings of this study.

The calibration curve is used to evaluate the consistency between the predicted risk of a predictive model and the actual risk. In this study, the constructed predictive model exhibited calibration curve slopes close to 1 in both the modeling group and validation group. This indicates that the model can accurately predict the risk of postoperative corneal edema in elderly patients with DC, showing a high level of consistency between the predicted outcomes and the actual occurrences. This has significant implications for clinicians in assessing preoperative surgical risks and devising personalized treatment plans. Receiver operating characteristic analysis is a commonly used method for evaluating the diagnostic performance of predictive models. By calculating the area under the curve, the model’s ability to distinguish between patients who will develop postoperative corneal edema and those who will not can be visually assessed. The study results indicate that the model demonstrated good predictive performance in both the modeling and validation groups, suggesting its effectiveness in identifying high-risk patients for postoperative corneal edema and providing a basis for clinical interventions. Lastly, DCA was used to evaluate the net benefit of the model in clinical decision-making. The DCA curve analysis in this study indicated that the model performed well in both the modeling group and validation group, providing good clinical benefits. This implies that using this predictive model for decision-making in clinical practice can bring more clinical benefits to patients, reducing unnecessary treatments and complications.

While this study has achieved certain outcomes, there are limitations to consider. First, while the sample size is adequate for an exploratory analysis, this study is inherently limited by its single-center, retrospective design, which may introduce selection bias and limit the statistical power for detecting rare confounders. To minimize potential bias during the selection process, we implemented strict and uniform inclusion/exclusion criteria (detailed in Section 2.1) before data collection. Consequently, the predictive model’s applicability to broader populations, particularly nondiabetic cataract patients or those from different geographic regions with varying ethnicities and healthcare practices, requires further validation. Future research should focus on multicenter, prospective studies with diverse cohorts to externally validate and refine the model’s transportability. Second, while we identified several key clinical factors, our analysis was necessarily constrained by the retrospective design. We were unable to account for other potential confounders that could influence corneal health and postoperative recovery, such as the use of systemic medications (e.g., anticoagulants, topical nonsteroidal anti-inflammatory drugs), detailed preoperative ocular surface health status (e.g., severity of dry eye, meibomian gland dysfunction), and patient adherence to postoperative anti-inflammatory regimens. The absence of these variables in our dataset represents a limitation. Future prospective studies should be designed to systematically collect and incorporate such factors. Exploring these additional variables could further refine the predictive accuracy of the model and provide a more holistic understanding of the pathophysiology of postoperative corneal edema.

## 5. Conclusion

In conclusion, this study analyzed the influencing factors of corneal edema in elderly patients with DC after phacoemulsification combined with intraocular lens implantation and constructed a predictive model with high predictive efficacy. The model demonstrates good calibration, discrimination, and clinical utility in clinical practices, providing valuable references for clinicians to reduce the incidence of postoperative corneal edema, enhance surgical outcomes, and improve patients’ quality of life. However, further research is needed to continuously refine and expand the applications of this model in the future.

## Author contributions

**Conceptualization:** Maierdanjiang Ainiwaer, Yingying Hong, Binghe Xiao, Li Ning, Yinghong Ji.

**Data curation:** Maierdanjiang Ainiwaer, Yingying Hong, Binghe Xiao, Li Ning, Yinghong Ji.

**Formal analysis:** Maierdanjiang Ainiwaer, Yingying Hong, Binghe Xiao, Li Ning, Yinghong Ji.

**Funding acquisition:** Maierdanjiang Ainiwaer, Yingying Hong, Yinghong Ji.

**Investigation:** Yingying Hong, Yinghong Ji.

**Writing – original draft:** Maierdanjiang Ainiwaer, Yingying Hong, Yinghong Ji.

**Writing – review & editing:** Maierdanjiang Ainiwaer, Yingying Hong, Yinghong Ji.
